# Fatal central nervous system co-infection with SARS-CoV-2 and tuberculosis in a healthy child

**DOI:** 10.1186/s12887-020-02308-1

**Published:** 2020-09-09

**Authors:** Bishara J. Freij, Bassam M. Gebara, Rabail Tariq, Ay-Ming Wang, John Gibson, Nidal El-Wiher, Graham Krasan, Paul M. Patek, Kelly A. Levasseur, Mitual Amin, Joseph M. Fullmer

**Affiliations:** 1 Beaumont Children’s Hospital, Beaumont Health, 3601 West 13 Mile Road, Royal Oak, MI 48073 USA; 2 Department of Neuroradiology, Beaumont Health, Royal Oak, MI USA; 3 Department of Pathology, Beaumont Health, Royal Oak, MI USA; 4 Department of Emergency Medicine, Beaumont Health, Royal Oak, MI USA

**Keywords:** SARS-CoV-2, CNS tuberculosis, Meningoencephalitis, Pediatric infections, Case report

## Abstract

**Background:**

Central and peripheral nervous system symptoms and complications are being increasingly recognized among individuals with pandemic SARS-CoV-2 infections, but actual detection of the virus or its RNA in the central nervous system has rarely been sought or demonstrated. Severe or fatal illnesses are attributed to SARS-CoV-2, generally without attempting to evaluate for alternative causes or co-pathogens.

**Case presentation:**

A five-year-old girl with fever and headache was diagnosed with acute SARS-CoV-2-associated meningoencephalitis based on the detection of its RNA on a nasopharyngeal swab, cerebrospinal fluid analysis, and magnetic resonance imaging findings. Serial serologic tests for SARS-CoV-2 IgG and IgA showed seroconversion, consistent with an acute infection. Mental status and brain imaging findings gradually worsened despite antiviral therapy and intravenous dexamethasone. Decompressive suboccipital craniectomy for brain herniation with cerebellar biopsy on day 30 of illness, shortly before death, revealed SARS-CoV-2 RNA in cerebellar tissue using the Centers for Disease Control and Prevention 2019-nCoV Real-Time Reverse Transcriptase-PCR Diagnostic Panel. On histopathology, necrotizing granulomas with numerous acid-fast bacilli were visualized, and *Mycobacterium tuberculosis* complex DNA was detected by PCR. Ventricular cerebrospinal fluid that day was negative for mycobacterial DNA. Tracheal aspirate samples for mycobacterial DNA and culture from days 22 and 27 of illness were negative by PCR but grew *Mycobacterium tuberculosis* after 8 weeks, long after the child’s passing. She had no known exposures to tuberculosis and no chest radiographic findings to suggest it. All 6 family members had normal chest radiographs and negative interferon-γ release assay results. The source of her tuberculous infection was not identified, and further investigations by the local health department were not possible because of the State of Michigan-mandated lockdown for control of SARS-CoV-2 spread.

**Conclusion:**

The detection of SARS-CoV-2 RNA in cerebellar tissue and the demonstration of seroconversion in IgG and IgA assays was consistent with acute SARS-CoV-2 infection of the central nervous infection. However, the cause of death was brain herniation from her rapidly progressive central nervous system tuberculosis. SARS-CoV-2 may mask or worsen occult tuberculous infection with severe or fatal consequences.

## Background

Except for the multisystem inflammatory syndrome in children, pandemic SARS-CoV-2 has been regarded as a milder respiratory and/or gastrointestinal infection in children, with lower rates of hospitalization and death as compared with adults [1–3]. Coronaviruses have been shown to be neuroinvasive in humans and animal models [4, 5]. In a retrospective study of 214 Chinese adults with COVID-19, many of whom had chronic underlying diseases, 78 (36.4%) exhibited neurologic symptoms including headache, dizziness, hyposmia, ischemic stroke, and cerebral hemorrhage [6]. In another series from France, 84% of 58 consecutive adults (median age, 63 years) admitted to the hospital with acute respiratory distress syndrome due to COVID-19 had neurologic symptoms such as agitation, corticospinal tract signs, and dysexecutive syndrome. MRI of the brain was performed in 13, of whom 8 had leptomeningeal enhancement and all exhibited perfusion abnormalities. Cerebrospinal fluid (CSF) analysis was only done in 7, and all had negative reverse transcriptase PCR assays for SARS-CoV-2 RNA [7]. Demonstration of SARS-CoV-2 RNA in the central nervous system (CNS) has only been documented in three adults, one with a positive CSF RNA result, one using genome sequencing in CSF, and another utilizing transmission electron microscopy on brain tissue obtained post-mortem [5, 8, 9]. Pulmonary co-infections with common viral, bacterial, and fungal pathogens have been described in COVID-19 patients, but there is a paucity of literature on mycobacteria, including members of the *Mycobacterium tuberculosis* complex with which a third of the world population is infected [10–17]. We describe the unique case of a previously healthy child with acute SARS-CoV-2 infection, fever, and headache, with laboratory and imaging evidence of meningoencephalitis who rapidly deteriorated 2 weeks into her illness and was found to have both SARS-CoV-2 RNA and *Mycobacterium tuberculosis* complex DNA on a cerebellar biopsy with histopathology consistent with tuberculous meningitis, with the recovery of *Mycobacterium tuberculosis* from two tracheal aspirates several weeks after she died. This report illustrates the need to consider that the toll of COVID-19-associated morbidity and mortality may not all be due to the virus.

## Case presentation

**Fig. 1:**
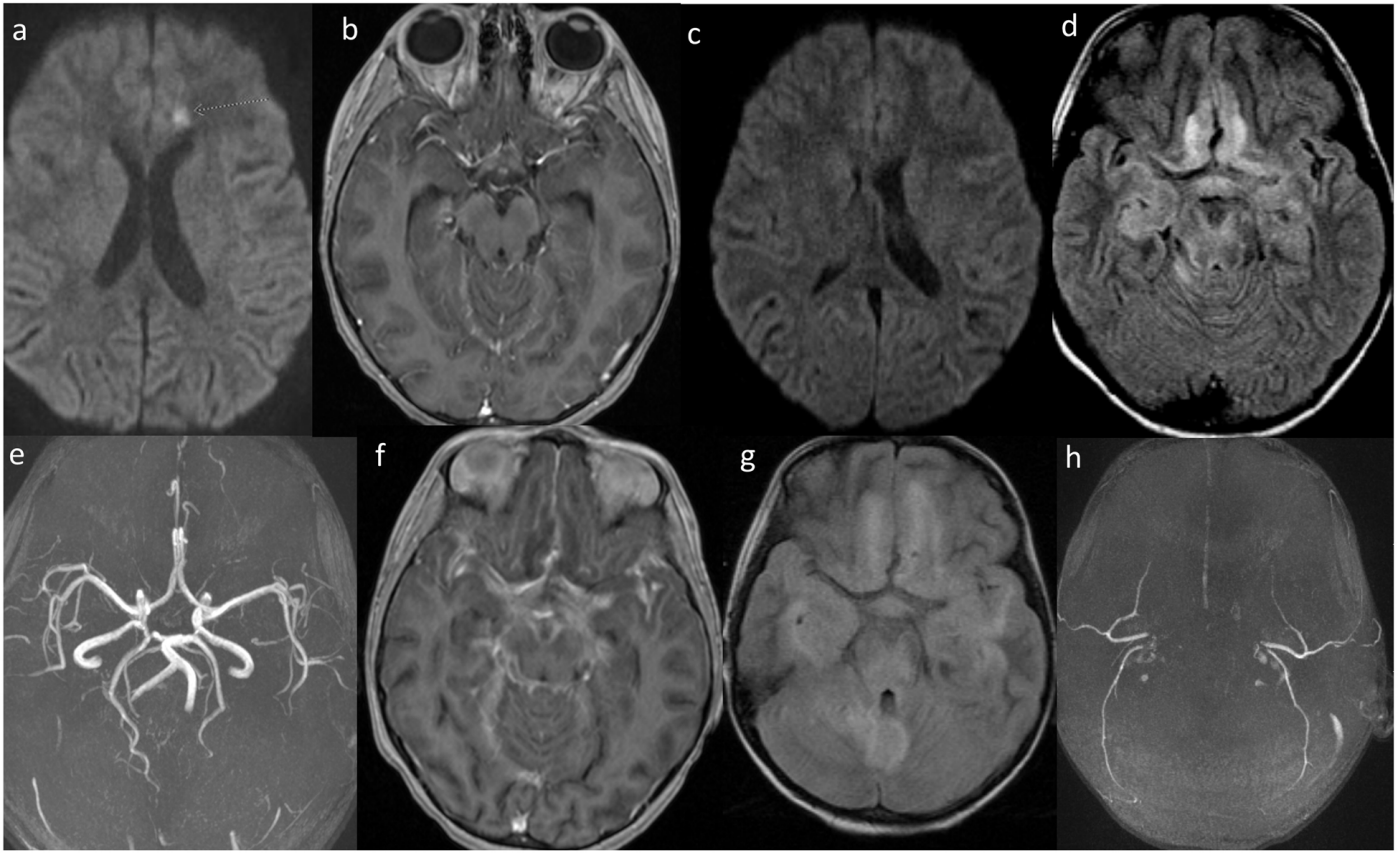
Selected sequential MRI/MRA images. **a** Diffusion-weighted image shows a 7 mm area of restriction diffusion in the left anterior frontal lobe subcortical white matter (arrow), day 12 of illness; **b** T1-weighted image with gadolinium showing mild leptomeningeal enhancement along the tentorium, day 12; **c** Diffusion-weighted image showing no restriction diffusion in the left anterior frontal lobe subcortical white matter, day 16; **d** T2 FLAIR shows abnormal increased signal mainly involving the midbrain, bilateral deep frontal, and bilateral medial temporal areas, day 16; **e** 3-D time of flight MRA shows a normal circle of Willis, day 16; **f** T1-weighted image with gadolinium showing diffuse leptomeningeal enhancement, day 16; **g** T2 FLAIR showing extensive grey matter lesions involving the deep frontal lobe, temporal lobe, midbrain, and cerebellar vermis, with no obvious cerebellar involvement, day 30; **h** 3-D time of flight MRA showing no flow in intracranial vessels with flow in extracranial vessels, day 30

A healthy five-year-old African American girl was admitted on the sixth day of fever (> 39 °C) and severe headache after 3 days of amoxicillin treatment for group A streptococcal pharyngitis. Initial laboratory data included a white blood cell count of 10.8 bil/L (lymphocytes 1.8 bil/L), Westergren sedimentation rate 30 mm/h, sodium 133 mmol/L, normal liver enzyme concentrations, and clear chest radiograph. Nasopharyngeal swab was positive for SARS-CoV-2 RNA using the NxTAG® CoV Extended Panel (Luminex). Her parents were recently ill with respiratory tract infections and SARS-CoV-2 IgG was positive after their recovery. Daily fever and headache persisted. A head CT scan on day 9 of illness was normal. She was discharged on day 10 but then readmitted later that evening following an episode of confusion and associated extremity stiffening with the appearance of staring into space, which lasted for 2 min. On readmission, CSF studies showed clear fluid with a white blood cell count of 24/μL (96% lymphocytes), red blood cell count 152/μL, protein 85 mg/dL (normal range, 15–45), and glucose 22 mg/dL (normal range, 50–80). Meningitis/encephalitis nucleic acid amplification panel using FilmArray® ME (Biofire) was negative for herpes simplex virus, human herpes virus VI (HHV-6), varicella-zoster virus, enterovirus, cytomegalovirus, parechovirus, *Cryptococcus neoformans/gatti*, *Escherichia coli* K1, *Haemophilus influenzae* type b, *Listeria monocytogenes*, *Neisseria meningitidis*, *Streptococcus agalactiae*, and *Streptococcus pneumoniae*. SARS-CoV-2 RNA was not detected in CSF using the Centers for Disease Control and Prevention 2019-nCoV Real-Time Reverse Transcriptase-PCR Diagnostic Panel (CDC qPCR). CSF bacterial culture was sterile. Her peripheral blood white blood cell count was 11.1 bil/L (lymphocytes 1.1 bil/L), platelet count 366 bil/L, sodium 130 mmol/L, ferritin 137 ng/mL, lactate dehydrogenase (LDH) 411 U/L, d-dimer 972 ng/mL, fibrinogen 316 mg/dL, troponin I < 0.01 ng/mL, and interleukin 6 level < 5 pg/mL. Oral hydroxychloroquine (6.5 mg/kg/dose every 12 h for two doses and then 3.5 mg/kg/dose per dose every 12 h for four more days) plus oral azithromycin 10 mg/kg/day for 5 days were started. Magnetic resonance imaging (MRI) of the brain with gadolinium on day 12 of illness revealed a 7 mm area of restriction diffusion involving the subcortical white matter of the medial aspect of the left anterior frontal lobe with increased T2 FLAIR signal without pathological enhancement (Fig. 1a). Mild leptomeningeal enhancement was noted (Fig. 1b). She developed SIADH (syndrome of inappropriate secretion of antidiuretic hormone) that was managed with fluid restriction and frequent 3% saline boluses. Her fever persisted but she was neurologically stable with waxing and waning cognition and headache, until day 15 when she became more lethargic and developed asymmetric pupils. Head CT scan showed enlargement of the lateral, third, and fourth ventricles. On day 16, repeat lumbar puncture showed an opening pressure of 35 cm of water. CSF studies revealed a hazy fluid with a white blood cell count of 160/μL (44% neutrophils, 51% lymphocytes, 5% monocytes), red blood cell count 7/μL, glucose 30 mg/dL, and protein 112 mg/dL; HHV-6 DNA was detected but not SARS-CoV-2 RNA. An external ventricular drain was placed the same day and the CSF was negative for both HHV-6 DNA and SARS-CoV-2 RNA. She had no HHV-6 DNA in plasma and her HHV-6 IgM was < 1:20 and HHV-6 IgG 1:320 (negative if < 1:10), suggesting that the detection of HHV-6 DNA on lumbar CSF was a false-positive result. MRI of the brain showed extensive progression of the meningoencephalitis to her cerebellum and corpus callosum, with leptomeningeal enhancement especially over the surface of the brainstem and into the auditory canals; magnetic resonance angiography (MRA) was normal (Figs. 1c-f). Intravenous dexamethasone was initiated. On day 17, she was intubated for worsening encephalopathy and intravenous remdesivir was started. Her chest radiograph showed early perihilar streaky opacities. Ferritin, interleukin 6, and LDH concentrations were again normal.

The subsequent clinical course over the ensuing days was turbulent. She had ongoing SIADH, severe hypertension requiring multiple intravenous antihypertensive medications, worsening chest radiographic findings with development of bibasilar opacities on day 27, electroencephalography consistent with severe encephalopathy, and gradual neurologic worsening with minimally reactive and dilated pupils.

On day 30 of illness, MRI of the brain showed marked progression of inflammation with supratentorial and infratentorial edema, hypoxic/ischemic changes, cerebellar tonsillar herniation, and lack of normal flow at the circle of Willis (Figs. 1g-h). A midline suboccipital craniectomy and C1 laminectomy were performed. The cerebellum was necrotic and edematous, and herniated out of the surgical defect. She passed away on day 32 of illness.

### Additional laboratory data

The absolute lymphocyte counts ranged between 0.4–1.8, and all were ≤ 1.0 during the last 2 weeks of her life. Her platelet count was normal throughout except for day 31 of illness (96 bil/L). AST, ALT, and total bilirubin concentrations between days 5 and 31 of illness (24 measurements each) were consistently normal.

Ventricular CSF studies from days 23 and 28 were similar, with clear fluid and white blood cell counts of 1–10/μL, red blood cell counts of 30–127/μL, glucose 75–83 mg/dL, and protein 53–55 mg/dL. On day 30, when she developed brain herniation, ventricular CSF was pale yellow with a glucose of 21 mg/dL, protein 130 mg/dL, white blood cell count 6/μL, and red blood cell count 174/μL.

Human immunodeficiency virus (HIV) types 1/2 antibodies and p24 antigen were negative. Antinuclear cytoplasmic antibody was undetectable.

Whole genome sequencing on blood did not reveal any abnormalities except for potential compound heterozygous *IL12RB* variants that may explain susceptibility to mycobacterial disease. Studies are planned to test parental DNA to identify whether these variants are on the same or different alleles (personal communication, July 20, 2020, Helen Su, M.D., Ph.D, National Institute of Allergy and Infectious Diseases, National Institutes of Health, Bethesda, Maryland).

Stored CSF from day 16 of illness was later tested for autoantibodies (CSF Autoimmune Evaluation, Mayo Clinic Laboratories, Rochester, MN). Immunofluorescence antibody assay detected glial fibrillary acidic protein (GFAP) autoantibody at a titer of 1:8 (normal, < 1:2).

### Microbiologic and immunologic studies

Nasopharyngeal swabs remained positive for SARS-CoV-2 RNA, including one collected on her last day of life. Tracheal aspirate SARS-CoV-2 RNA was negative on day 22.

Tracheal aspirates for mycobacterial DNA detection and cultures were collected on days 22 and 27 of illness. *Mycobacterium tuberculosis* complex DNA was negative for both samples; however, they grew acid fast bacilli after 8 weeks, several weeks after the child’s demise. The organism was later identified as *Mycobacterium tuberculosis* by the Michigan Department of Health and Human Services, and it was susceptible to isoniazid, rifampin, ethambutol, and pyrazinamide. Mycobacterial culture from ventricular CSF collected on day 25 was sterile. Mycobacterial DNA detection on ventricular CSF from day 28 of illness was negative.

The cerebellar brain biopsy was positive for SARS-CoV-2 RNA (CDC qPCR). It was also positive for *M*. *tuberculosis* complex DNA using an hsp65 amplified probe and negative for atypical mycobacterial DNA with 16 s rDNA, hsp65, and rpoB primer sets (University of Washington Medical Microbiology Laboratories, Seattle, WA). There was no fresh cerebellar tissue for mycobacterial culture.

**Fig. 2:**
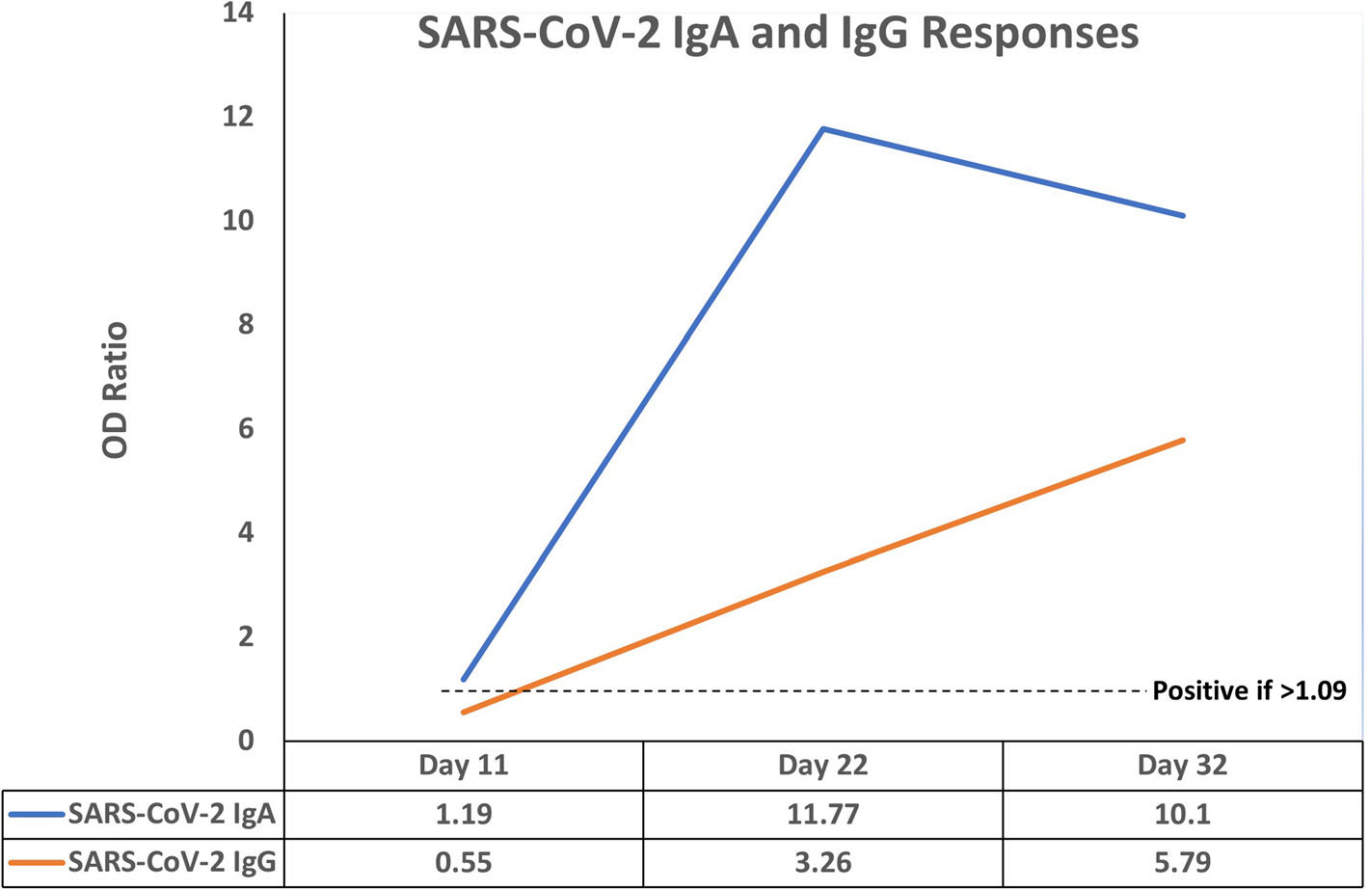
Evolution of SARS-CoV-2 IgA and IgG responses by enzyme immunoassay (EUROIMMUN AG, Germany). OD = Optical density

SARS-CoV-2 IgA and IgG responses on day 11 of illness were weakly positive and negative, respectively. A strong antibody response evolved when re-tested on days 22 and 32 (Fig. 2). The findings were consistent with an acute SARS-CoV-2 infection.

The child had no history of travel and lived with her mother, father, and maternal grandmother, all of whom were positive for SARS-CoV-2 IgG but had negative interferon-γ release assays and normal chest radiographs. She was also in frequent contact with her maternal aunt’s family, consisting of 3 adults, all of whom had normal chest radiographs and negative SARS-CoV-2 IgG and interferon-γ release assays. Schools had closed in Michigan because of the COVID-19 pandemic a week before her symptoms began. The source of her tuberculous infection has not been identified.

### Histopathology

**Fig. 3:**
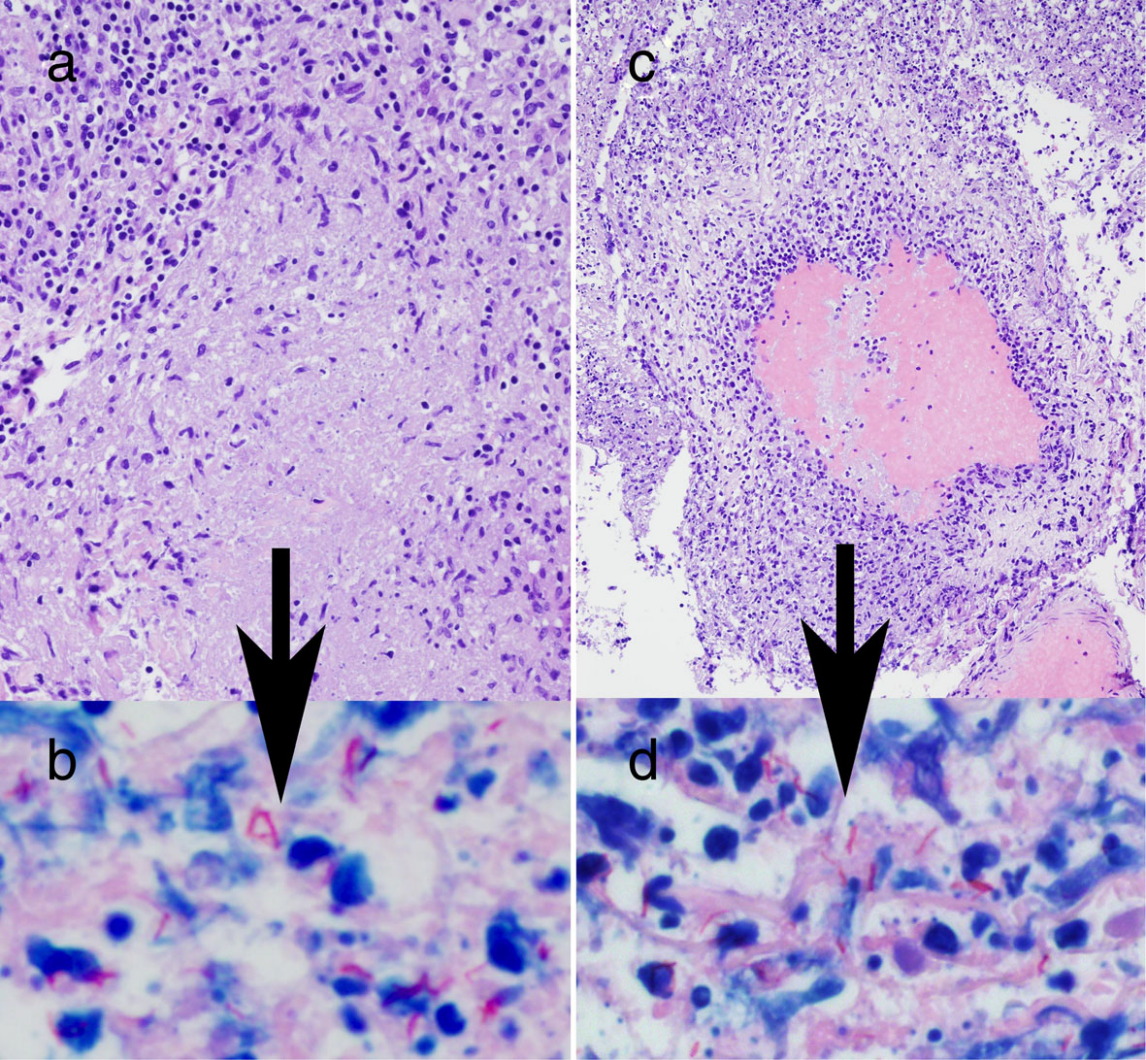
Cerebellar biopsy histopathology. **a** High power view of a necrotizing granuloma showing central pale eosinophilic caseous-type necrosis; **b** High power view revealing numerous acid-fast bacilli within the granuloma; **c** Low power view of medium-sized blood vessel with numerous inflammatory cells and severe damage to the vessel wall with essentially complete loss of internal elastic lamina (vasculitis); **d** High power view revealing numerous acid-fast bacilli identified in same vessel wall. Hematoxylin-Eosin stained sections (**a**, **c**); Fite stain (**b**, **d**)

The cerebellar biopsy on day 30 of illness showed patchy areas of necrotizing granulomatous inflammation and vasculitis. Stains showed endothelial injury, muscle wall injury, and lost elastic lamina. Acid fast bacilli were identified (Fig. 3).

## Discussion and conclusions

SARS-CoV-2 neurotropism is believed to be mediated by binding of its spike protein to its receptor angiotensin-converting enzyme 2 which is expressed in glial cells and neurons [18]. Neurologic abnormalities are increasingly recognized in SARS-CoV-2 infected individuals, but our knowledge is hampered by a dearth of information on associated CSF and neuroradiologic correlates of pathology and how they might evolve during a severe illness. Contributing to this knowledge gap are resource limitations including insufficient reagents for broader molecular testing and infection control concerns that limit diagnostic evaluations such as imaging studies, tissue biopsies, and autopsies. Severe or fatal illnesses are generally attributed to SARS-CoV-2, which may be inaccurate.

This child’s clinical course was that of progressive clinical and radiologic worsening. She was shown to have an acute SARS-CoV-2 infection based on evolution of her serologic responses (Fig. 2). The CSF abnormalities were consistent with acute viral meningoencephalitis, and similar findings have been described in two adults with SARS-CoV-2-associated encephalitis [19]. However, her initial CSF glucose was concerning because it was unusually low for a viral infection. While tuberculous meningitis was considered during the course of her illness, it was deemed improbable given the lack of known exposures to tuberculosis (later confirmed by evaluation of all family members who proved to be free from infection), normal chest radiographs during the first 2 weeks of illness, and CSF findings that were uncommon for this diagnosis (commonly white blood cell counts of 100–500/μL and protein > 100 mg/dL) [20]. Tracheal aspirate and CSF molecular tests were negative for mycobacteria. Nevertheless, normal chest radiographs in children with tuberculous meningitis are commonly encountered and are not sufficient to exclude the diagnosis [21]. In the pre-COVID-19 era, the diagnosis of tuberculous meningitis would have been considered possible and specific anti-tuberculous therapy empirically initiated [22].

We hypothesize that this child was asymptomatically infected with *Mycobacterium tuberculosis* when she acquired her SARS-CoV-2 infection. SARS-CoV-2 meningoencephalitis was confirmed by the positive cerebellar tissue PCR assay. The immunologic response to SARS-CoV-2 may have contributed to CNS spread of mycobacteria, as detailed below. The cause of death was brain herniation secondary to rapidly progressive CNS tuberculosis.

Type I interferons (e.g., IFN-α, IFN-β) are produced early during a viral infection and are critical in antiviral immunity. After attachment to the interferon α/β receptor and ensuing intermediary biochemical steps, interferon stimulated genes (ISGs) are activated [23]. Wei and colleagues demonstrated that genes functioning in viral defense, such as ISGs, are highly expressed in peripheral blood mononuclear cells of SARS-CoV-2 infected individuals requiring intensive care [24]. In one study, peak IFNα2 levels occurred on days 8–10 of symptom onset and gradually decreased over the following 2–3 weeks in critically ill adults [25].

Multiple studies have shown that viruses such as influenza, HIV, and measles can impair the ability of macrophages to contain mycobacterial growth, including *Mycobacterium tuberculosis* [26, 27]. In a mouse model of influenza A and *M*. *tuberculosis*, co-infection resulted in enhanced mycobacterial growth in the lungs and decreased survival mediated by type I IFN signaling [28]. Type I interferons also reduce the ability of macrophages to respond to IFN-γ and control of intracellular growth of *M*. *tuberculosis* [29]. This child additionally had persistent lymphopenia, which is a risk factor for severe tuberculosis [30].

The detection of a high CSF GFAP autoantibody titer is indicative of an astrocytopathy. This autoantibody is often detected in individuals with meningoencephalitis [31]. Severe SIADH has previously been observed in a few adults with SARS-CoV-2 pneumonia [32].

This child’s fatal illness raises broader concerns given the intersection of a global SARS-CoV-2 pandemic and an infection that afflicts a third of the world population. He and colleagues described 2 adults with a history of treated tuberculosis and one with a 50-year history of untreated but stable infection who acquired SARS-CoV-2 infection and had severe pulmonary disease with protracted hospital stays, but survived; all were placed on anti-tuberculous drug therapy, but no microbiologic or molecular studies for *Mycobacterium tuberculosis* were attempted [12]. A number of other studies have examined small cohorts of adults with known tuberculosis and COVID-19 superinfection, all suggesting that those already on anti-tuberculous therapy may have a relatively benign course [14], although there is some evidence to the contrary [15]. In terms of tuberculous meningitis, we found a report of two adults who were on treatment but acquired COVID-19 53–152 days after their diagnosis. One had worsening chest radiographs and the other had improved imaging findings when compared to their last pre-COVID-19 studies; both survived [14]. A three-month-old Gambian child with COVID-19 was diagnosed with pulmonary and extrapulmonary tuberculosis (including meningitis) 3 days later and was placed on anti-tuberculous medications, but no details of how the diagnosis was reached were provided [16]. Further investigation into the role of co-infection by *Mycobacterium tuberculosis* and SARS-CoV-2 on severity and mortality is needed for both adults and children.

## Data Availability

Not applicable as no datasets were generated or analyzed for the current case report. Patient data are part of the protected electronic health system.

## Abbreviations

CDC qPCR: Centers for Disease Control and Prevention 2019-nCoV Real-Time Reverse Transcriptase-PCR Diagnostic Panel
CNS: Central nervous system
CSF: Cerebrospinal fluid
CT: Computed tomography
HIV: Human immunodeficiency virus
IFN: Interferon
ISGs: Interferon stimulated genes
